# Probing prothrombin structure by limited proteolysis

**DOI:** 10.1038/s41598-019-42524-z

**Published:** 2019-04-16

**Authors:** Laura Acquasaliente, Leslie A. Pelc, Enrico Di Cera

**Affiliations:** Edward A. Doisy Department of Biochemistry and Molecular Biology, Saint Louis University School of Medicine, St. Louis, MO 63104 USA

## Abstract

Prothrombin, or coagulation factor II, is a multidomain zymogen precursor of thrombin that undergoes an allosteric equilibrium between two alternative conformations, open and closed, that react differently with the physiological activator prothrombinase. Specifically, the dominant closed form promotes cleavage at R320 and initiates activation along the meizothrombin pathway, whilst the open form promotes cleavage at R271 and initiates activation along the alternative prethrombin-2 pathway. Here we report how key structural features of prothrombin can be monitored by limited proteolysis with chymotrypsin that attacks W468 in the flexible autolysis loop of the protease domain in the open but not the closed form. Perturbation of prothrombin by selective removal of its constituent Gla domain, kringles and linkers reveals their long-range communication and supports a scenario where stabilization of the open form switches the pathway of activation from meizothrombin to prethrombin-2. We also identify R296 in the A chain of the protease domain as a critical link between the allosteric open-closed equilibrium and exposure of the sites of cleavage at R271 and R320. These findings reveal important new details on the molecular basis of prothrombin function.

## Introduction

**Figure 1:**
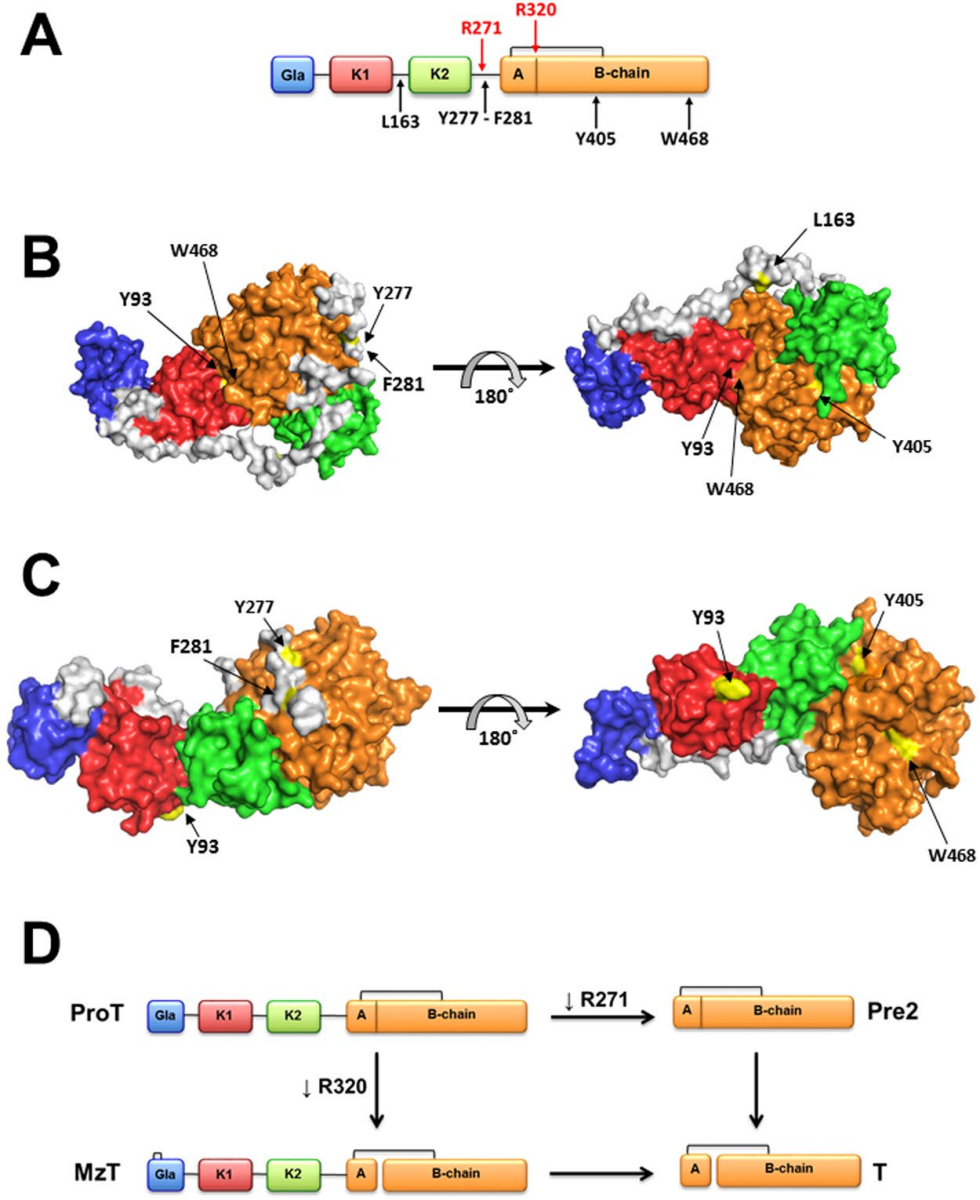
(**A**) Schematic representation of prothrombin with its constituent Gla domain, kringles (K1 and K2) and protease domain containing the A and B chain. The sites of activation, R271 and R320, are labeled in red. Sites of proteolytic attack by chymotrypsin are labeled in black. X-ray crystal structures of the closed (**B**, PDB ID: 6C2W) and open (**C**, PDB ID: 5EDM) forms of prothrombin that interconvert by exploiting the flexibility of linker regions like Lnk2. The open form is attacked by chymotrypsin at W468 in the autolysis loop. The closed form is protected from this cleavage by the collapse of Y93 in kringle-1 into the active site where it engages W468 and W547 in stacking interactions. (**D**) Schematic representation of the four intermediates of the conversion of prothrombin (ProT) to thrombin (T). The conversion involves cleavage at R271 and R320. The former cleavage sheds the auxiliary Gla domain and kringles and generates the inactive intermediate prethrombin-2 (Pre2). The latter cleavage separates the A and B chain, that remain connected through the C293-C439 disulfide bond, and generates the active intermediate meizothrombin (MzT).

The response of the body to vascular injury entails activation of a cascade of proteolytic events where zymogens are converted into active proteases^1^. In the penultimate step of this cascade, the zymogen prothrombin is converted to the active protease thrombin in a reaction catalyzed by the prothrombinase complex composed of the enzyme factor Xa, cofactor Va, Ca^2+^ and phospholipids. Prothrombin is one of the most abundant proteins circulating in the blood and features a modular assembly that includes the N-terminal Gla domain (residues 1–46), kringle-1 (residues 65–143), kringle-2 (residues 170–248) and a C-terminal protease domain (residues 285–579). Completing the architecture are three linker regions connecting the Gla domain to kringle-1 (Lnk1, residues 47–64), the two kringles (Lnk2, residues 144–169) and kringle-2 to the protease domain (Lnk3, residues 249–284). Prothrombinase cleaves prothrombin at two sites, i.e., R271 in Lnk3 and R320 in the protease domain. Cleavage at R271 sheds the auxiliary domains and generates the inactive intermediate prethrombin-2. The alternative cleavage at R320 separates the A (residues 285–320) and B (residues 321–579) chains in the protease domain, that remain connected through the C293-C439 disulfide bond, and yields the active intermediate meizothrombin correctly folded for catalysis (Fig. 1). On the surface of platelets^2,3^, prothrombinase activates prothrombin along the prethrombin-2 pathway^3^. On non-platelet surfaces such as red blood cells^4^ or synthetic phospholipids^5–7^ most often used for *in vitro* studies, prothrombinase activates prothrombin along the meizothrombin pathway. Factors that directs prothrombinase to cleave at R271 or R320 have been identified^7–11^. For example. perturbations of the Gla domain switch the pathway of activation from meizothrombin to prethrombin-2^7,12,13^ and so does active site occupancy^14,15^. Structural context for the mechanism of activation has emerged recently from X-ray studies of prothrombin^16–18^. The modular assembly of prothrombin produces a plastic fold subject to an allosteric equilibrium between closed and open forms. The domains align vertically in the open form (Fig. 1) and preferentially present R271 for cleavage to prothrombinase. The closed form predominates in solution and features a collapse of kringle-1 into the active site of the protease domain (Fig. 1), with a concomitant preferential exposure of R320 for cleavage by prothrombinase. Therefore, the open and closed forms initiate activation along the prethrombin-2 or meizothrombin pathways, respectively^16^. This simple partitioning between open and closed forms explains several previous observations on prothrombin activation^7–11^ and offers a starting point for additional investigation. Unraveling the molecular cross-talk among distinct domains of prothrombin in the context of the allosteric open-closed equilibrium becomes of interest.

In this study, we investigate prothrombin allostery by exploiting the unique properties of this zymogen in its interaction with chymotrypsin. The open form of prothrombin is readily attacked by chymotrypsin at W468 in the flexible autolysis loop of the protease domain, as observed for thrombin^17^, but the closed form is resistant to proteolysis because of the intramolecular collapse of residue Y93 onto residue W547 shaping the west wall of the active site in the protease domain (Fig. 1). Hence, the open-closed equilibrium is directly linked to accessibility of the autolysis loop in the protease domain and limited proteolysis by chymotrypsin becomes useful to interrogate individual domains of prothrombin in their contribution to the allosteric equilibrium and mechanism of activation.

## Results

### Prothrombin derivatives and their susceptibility to chymotrypsin proteolysis

**Figure 2:**
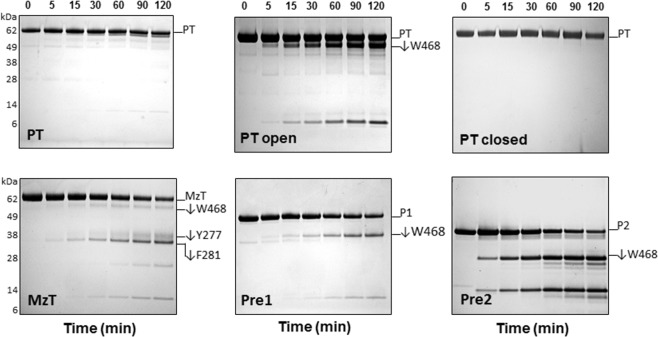
Exposure of the autolysis loop in the components of prothrombin activation as probed by limited proteolysis with chymotrypsin. After conversion of prothrombin (PT) to the active meizothrombin (MzT) or inactive prethrombin-1 (Pre1) and prethrombin-2 (Pre2) derivates, the autolysis loop becomes exposed and susceptible to chymotrypsin attack at W468. In the case of MzT, chymotrypsin cleaves additionally at Y277 and F281 in Lnk3, generating a 38 kDa species. Exposure of the autolysis loop reflects the open-closed equilibrium of prothrombin. The open conformation (mutant Y93A) is proteolytically attacked by chymotrypsin, but the closed form (mutant S101C/A470C) is not. All reactions were conducted in TBS buffer at 37 °C and analyzed by SDS-PAGE under non-reducing conditions. Chemical identities of the bands were verified by N-terminal sequencing (see Supplementary Table S1). None of the gels were cropped and originals are available in the Supplementary Information as Fig. S2.

The two forms of prothrombin, open and closed, are selectively stabilized by mutations that either abrogate the intramolecular interaction between kringle-1 and the protease domain (Y93A)^17^ or cross-link these two domains (S101C/A470C)^16^. Limited proteolysis by chymotrypsin reveals that the closed form is resistant to proteolysis, as seen for wild-type, but the open form is readily accessible to cleavage at W468 (Fig. 2). The results confirm that wild-type prothrombin exists predominantly in the closed form, as concluded from single molecule measurements^17^. Activation of prothrombin by prothrombinase generates the intermediates meizothrombin and prethrombin-2 upon cleavage at R320 and R271, respectively (Fig. 1). Meizothrombin is readily attacked at W468 in the autolysis loop and additionally at Y277 and F281 in Lnk3 (Fig. 2), which is consistent with the allosteric open-closed equilibrium of this intermediate being switched to the open form^16^. Likewise, prethrombin-2 is readily cleaved by chymotrypsin at W468 because removal of the auxiliary Gla domain and kringles in this intermediate no longer enables the intramolecular collapse necessary to stabilize the closed form (Fig. 1). Prethrombin-1 generated from prothrombin upon cleavage by factor Xa at R155 in Lnk1 is also susceptible to proteolysis by chymotrypsin, which is explained by removal of the Gla domain, Lnk1 and kringle-1 in this intermediate.

### Effect of binding to prothrombinase

The data in Fig. 2 are consistent with a scenario where prothrombin exists predominantly in a closed conformation characterized by the intramolecular collapse of kringle-1 onto the protease domain and switches to the open conformation when the collapse is removed after cleavage at R320 and generation of meizothrombin. Because the relative distribution of closed and open conformations changes when prothrombin transitions to meizothrombin, it is likely that prothrombinase exists in multiple conformations that accommodate changes in the multidomain architecture of its substrates. This reciprocal plasticity between enzyme (prothrombinase) and substrate (prothrombin, meizothrombin) explains the significant context dependence of prothrombin activation along the meizothrombin or prethrombin-2 pathways^2–7^.

### Role of auxiliary domains and linkers

**Figure 3:**
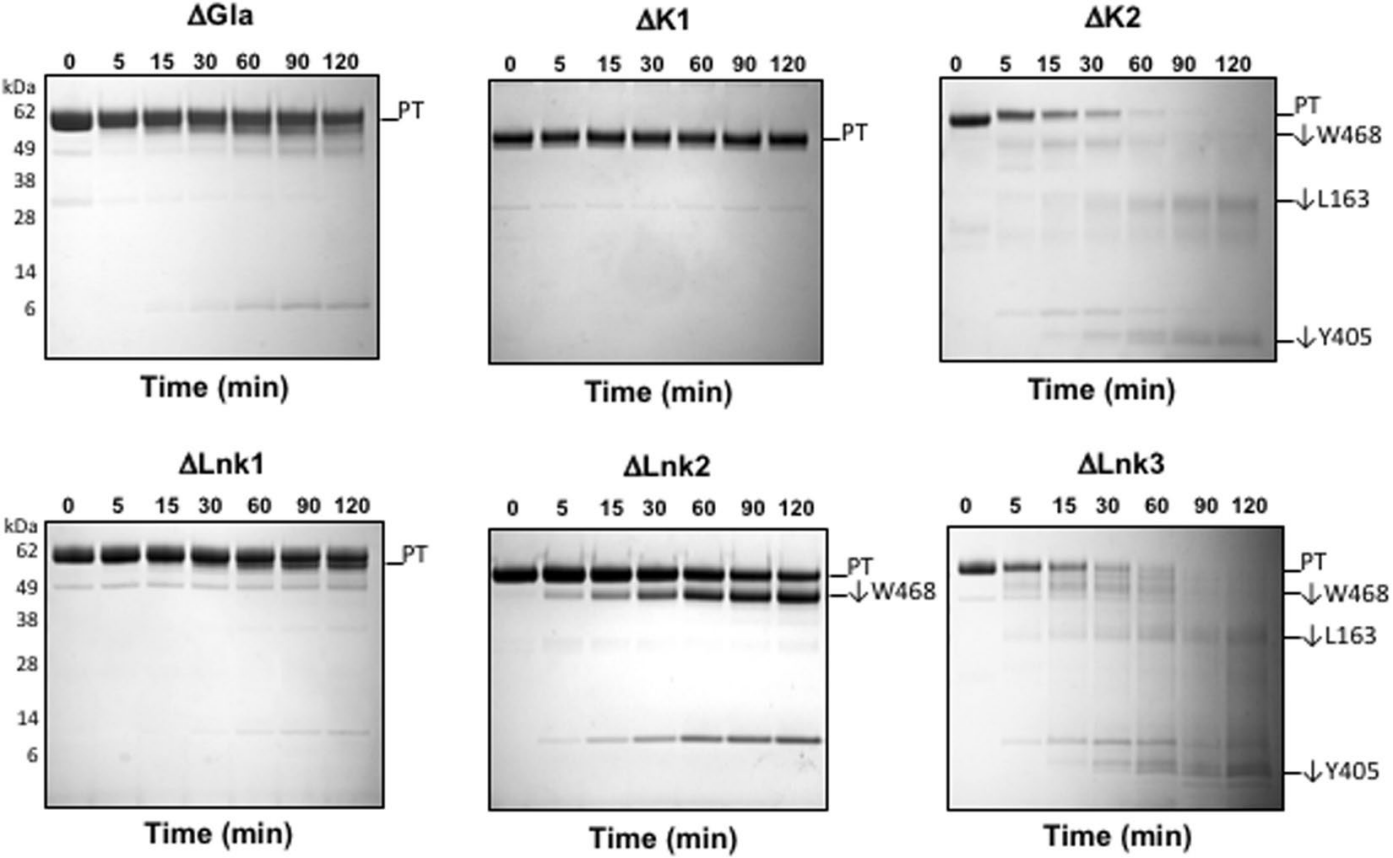
Effect of auxiliary domains on prothrombin proteolysis by chymotrypsin. Chymotrypsin cleaves at W468 in the autolysis loop only when prothrombin assumes the open conformation (ΔLnk2, ΔLnk3; see also Fig. 2B). Upon removal of kringle-2 (ΔK2) and Lnk3 (ΔLnk3), chymotrypsin proteolysis leads to accumulation of 50 and 6 kDa species, due to the cleavage at W468, and two major bands at 30 and 5 kDa resulting from cleavage at L163 in Lnk2 and Y405 in the protease domain (see also Fig. 1C). Removal of the Gla domain (ΔGla), kringle-1 (ΔK1) or Lnk1 (ΔLnk1) makes prothrombin resistant to proteolytic attack. Chemical identities of the bands were verified by N-terminal sequencing. None of the gels were cropped and originals are available in the Supplementary Information as Fig. S3.

**Figure 4:**
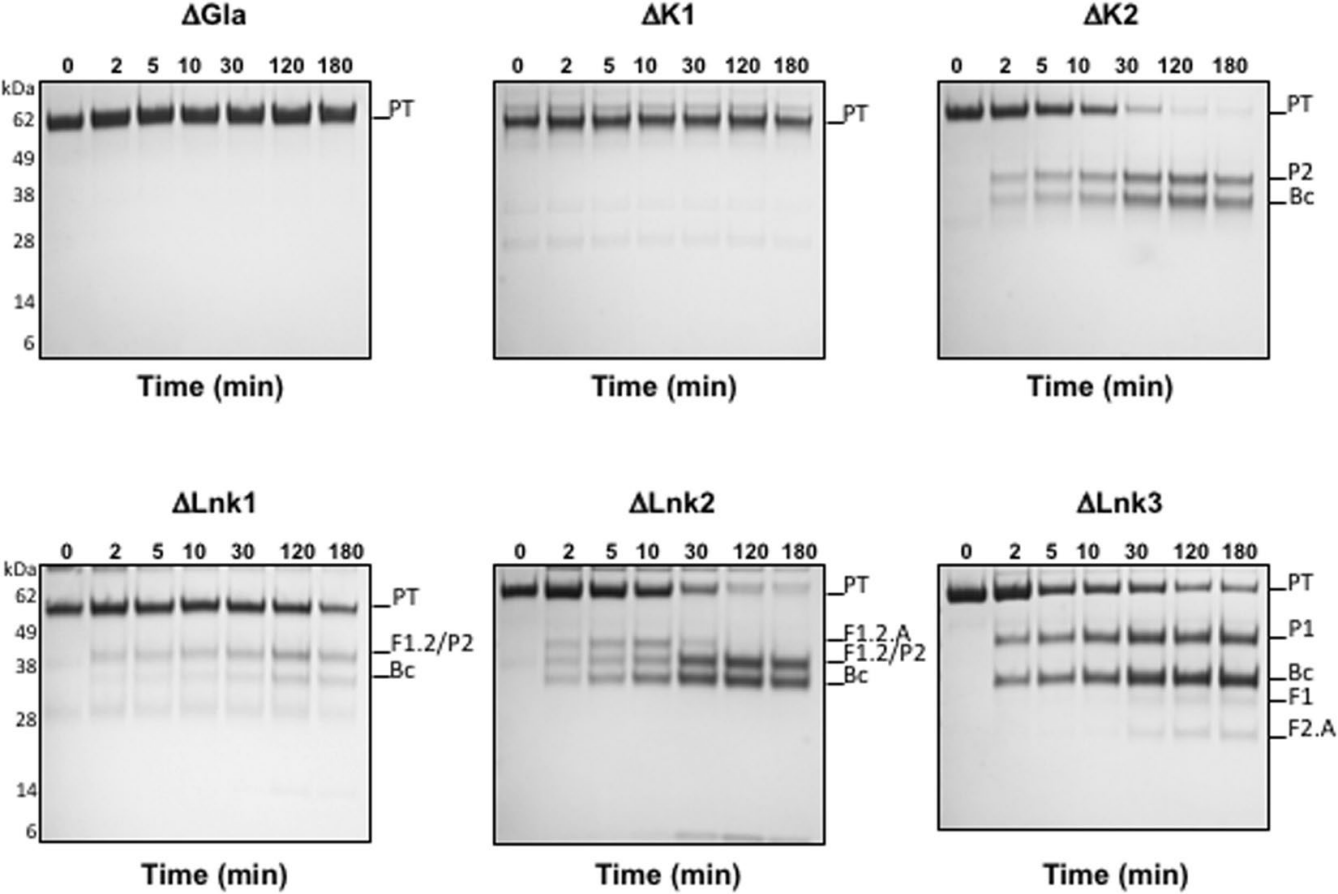
Effect of removal of individual domains and linkers on prothrombin activation by prothrombinase. Prothrombin wild-type is cleaved by prothrombinase at R320 along the meizothrombin pathway to generate fragment-1.2.A and the B-chain (Fig. 4C). Prothrombin ΔLnk2 is activated along both the meizothrombin and prethrombin-2 pathways. Prothrombin ΔLnk1 and ΔK2 follow exclusively the prethrombin-2 (P2) pathway, with formation of prethrombin-2 after cleavage at R271. In prothrombin ΔLnk3, residue R271 is missing and activation proceeds by formation of prethrombin-1 (P1). Notably, under the same experimental conditions, prothrombin derivatives ΔGla and ΔK1 show no detectable specificity for prothrombinase. All reactions were conducted in TBS buffer at 37 °C, with the inhibitor DAPA added to abrogate activity for thrombin and meizothrombin. Chemical identities of the bands were verified by N-terminal sequencing. None of the gels were cropped and originals are available in the Supplementary Information as Fig. S4.

Additional details on the conformational properties of prothrombin are revealed by removal of individual domains and linkers (Fig. 3). It should be stressed that these perturbations are unlikely to introduce anomalous perturbations of the prothrombin fold. Many of the derivatives have been crystallized (ΔGla^19^, prethrombin-1 as a combination of ΔGla, ΔLnk1 and ΔK1^20^, ΔLnk2 in various deletions^18,21^, prethrombin-2 as a combination of ΔGla, ΔLnk1, ΔK1, ΔLnk2, ΔK2 and ΔLnk3^22,23^) and have architectures consistent with current crystal structures of prothrombin^17–19,21^. Limited proteolysis by chymotrypsin partitions deletions in two groups based on their position along the prothrombin sequence. Removal of the N-terminal Gla domain, Lnk1 or kringle-1 produces derivatives that are resistant to proteolysis, whilst removal of the C-terminal Lnk2, kringle-2 or Lnk3 produces rapid attack at W468 and other sites. Interestingly, the proteolytic profile generated upon removal of Lnk3 is similar to that obtained upon removal of kringle-2, vouching for some structural determinants in Lnk3 that help stabilize the interaction of kringle-2 with the protease domain and communicate long range with the autolysis loop. The sharp partitioning in the proteolytic profiles in Fig. 3 suggests that deletion of N-terminal auxiliary domains leaves prothrombin in the closed form and deletion of C-terminal auxiliary domains switches the zymogen to the open form. Strong support to this conclusion comes from inspection of the pathway of activation of the C-terminal deletions, that proceeds along the prethrombin-2 intermediate (Fig. 4). Interestingly, removal of Lnk2 produces activation along both the meizothrombin and prethrombin-2 pathways as though deletion of this linker “freezes” the open-closed equilibrium. Indeed, Lnk2 confers flexibility to prothrombin by allowing the rigid Gla domain/kringle-1 pair to assume multiple orientations relative to the rigid kringle-2/protease domain pair^18,21^. The data in Figs 3 and 4 also prove that removal of Lnk3 stabilizes the open form, although this linker should not affect the rigidity of the kringle-2/protease domain interface first documented in the structure of prethrombin-1^20^ and later confirmed in all structures of prothrombin^16–18^. The similarity of proteolytic profiles between removals of Lnk3 and kringle-2 suggests that the two domains are strongly linked and may produce similar structural perturbations. The correlation between deletion of auxiliary components and pathway of activation is less obvious in the case of N-terminal segments (Fig. 4). Deletion of Lnk1 switches the pathway to prethrombin-2, despite its resistance to chymotrypsin proteolysis (Fig. 3). The paradox can be explained by the fact that Lnk1 and the adjacent kringle-1 are important epitopes for cofactor Va binding^24,25^ that is required for activation to proceed along the meizothrombin pathway^5^. Deletion of the Gla domain or kringle-1 is too disruptive for prothrombinase binding to establish the pathway of activation under the conditions of the assay (Fig. 4). The various constructs also affect the rate of prothrombin activation by prothrombinase. Values of the specificity constant ${k}_{{cat}}/{K}_{m}$ are summarized in Table 1 and are perturbed mostly through ${k}_{{cat}}$ upon removal of the Gla domain, kringle-2 and Lnk3. The Gla domain is necessary for efficient interaction with phospholipids^5^, kringle-2 contains epitopes for interaction with factor Xa^26^ and cofactor Va^24,25^ and Lnk3 contains the site of cleavage at R271. Again, the similarity between removal of Lnk3 and kringle-2 supports a strong functional linkage between the two structural determinants that likely extends to presentation of the sites of activation to prothrombinase.

**Table 1:** Kinetic rate constants for activation of prothrombin constructs by prothrombinase.

Prothrombin	*k*_*cat*_/*k*_*m*_ (μM^−1^s^−1^)	*k*_*cat*_ (s^−1^)	*k*_*m*_ (μM)
wild-type	228 ± 9	30 ± 2	0.13 ± 0.01
ΔGla	0.49 ± 0.01	0.38 ± 0.01	0.77 ± 0.03
ΔK1	62 ± 3	12 ± 1	0.19 ± 0.01
ΔK2	10 ± 3	3.3 ± 0.2	0.32 ± 0.02
ΔLnk1	106 ± 5	17 ± 3	0.16 ± 0.01
ΔLnk2	110 ± 3	9.7 ± 0.9	0.087 ± 0.002
ΔLnk3	7.3 ± 0.8	1.5 ± 0.1	0.20 ± 0.01
R296A	16 ± 2	9.4 ± 0.1	0.64 ± 0.03

Data were obtained from analysis of progress curves of prothrombin activation as reported elsewhere^18^. Experiments were carried out in TBS buffer at 37 °C.

### Role of residue R296 in selecting the pathway of prothrombin activation

**Figure 5:**
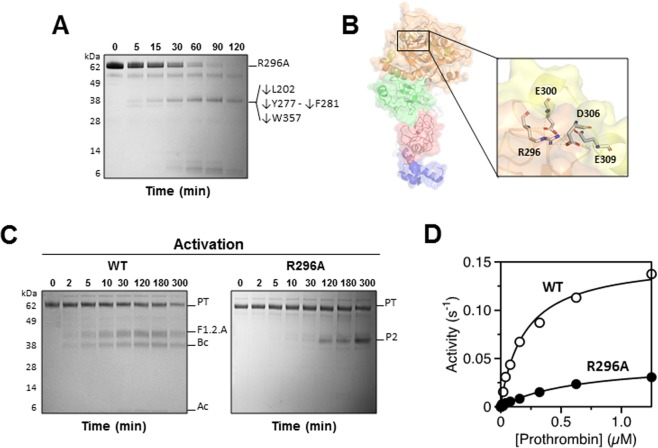
Critical role of residue R296 in the allosteric open-closed equilibrium and activation of prothrombin. (**A**) Chymotrypsin efficiently digests prothrombin R296A yielding a ~38 kDa specie after cleavage at Y277 and F281 in Lnk3, L202 in kringke-2 and W357 in the protease domain. (**B**) Residue R296 is located towards the N-terminal of the A-chain (yellow, PDB ID: 5EDM) of the protease domain where it is involved in the R296-E300-D306-E309 ionic cluster. (**C**) Activation of prothrombin R296A by the prothrombinase complex monitored by SDS-PAGE under reducing conditions indicates the generation of prethrombin-2 (P2, 38 kDa). Under the same experimental conditions, prothrombin wild-type is first cleaved at R320 producing fragment-1.2.A (40 kDa) and the B chain of thrombin (30 kDa), and then at R271 with generation of the smaller A chain (6 kDa). The R296A mutation switches the pathway of activation from meizothrombin to prethrombin-2 and significantly reduces the amount of thrombin activity generated upon interaction with the physiological activator prothrombinase. All reactions were conducted in TBS buffer at 37 °C. Chemical identities of the bands were verified by N-terminal sequencing. None of the gels were cropped and originals are available in the Supplementary Information as Fig. S5. (**D**) Michaelis-Menten plot of the initial rates of prothrombin cleavage by prothrombinase plotted versus the concentration of prothrombin for wild-type (WT) and mutant R296A. The rates measure the activity of thrombin toward the chromogenic substrate FPR-pNA after generation from prothrombin.

Additional evidence of structural and functional communication between Lnk3 and kringle-2 comes from the intriguing properties of the single site mutation R296A that has a profound influence on the conformation of prothrombin and its mechanism of activation (Fig. 5). Residue R296 is located in the A chain of the protease domain where it participates in ionic interactions with the acidic residues E300, D306 and E309. Replacement of E300 and E309 with Lys in the naturally occurring prothrombins Denver is associated with severe hemophilia-like bleeding^27^. A systematic mutagenesis study of charged residues of the A chain of thrombin identified R296 as critical to the conversion of prethrombin-1 to thrombin by prothrombinase, but the molecular origin of the effect was not investigated^28^. The R296A mutation changes the conformation of prothrombin that becomes susceptible to chymotrypsin proteolysis at multiple residues other than W468, i.e., L202 in kringle-2, Y277 and F281 in Lnk3 and W357 in the protease domain (Fig. 5). The proteolytic profile shows rapid attack of prothrombin as for removal of Lnk3 or kringle-2 (Fig. 3), which suggests that R296 plays a structural role in stabilizing the rigid interface between kringle-2 and the protease domain. Importantly, under experimental conditions where wild-type is activated by prothrombinase along the meizothrombin pathway, identified under reducing conditions by the appearance of fragment-1.2.A (residues 1–320, 40 kDa) and the B chain of the protease domain (residues 321–579, 30 kDa), the R296A mutant is converted to thrombin along the alternative prethrombin-2 pathway (Fig. 5). The switch in the pathway of activation produces accumulation of the inactive intermediate prethrombin-2 and drops the ${k}_{{cat}}/{K}_{m}$ value for thrombin generation > 10-fold compared to wild-type (Table 1). The perturbation is comparable to that seen upon removal of Lnk3 or kringle-2 and explains the severe bleeding phenotype of prothrombins Denver^27^.

## Discussion

Recent structural studies have identified two major conformations of prothrombin, open and closed, differing in the accessibility of the active site and the overall arrangement of its domains^16–18^. The two conformations are associated with the two distinct pathways of activation, with the closed one promoting cleavage at R320 and initiating the meizothrombin pathway and the open one promoting cleavage at R271 and initiating the alternative prethrombin-2 pathway. It has been known for a long time that the pathway of prothrombin activation depends on the context. On the surface of platelets^2,3^, prothrombinase activates prothrombin along the prethrombin-2 pathway. On non-platelet surfaces such as red blood cells^4^ or synthetic phospholipids^5–7^, prothrombinase activates prothrombin along the meizothrombin pathway. Direct evidence that prothrombin may switch its conformation from closed to open according to the context is currently not available, nor is there information on which conformation prothrombin assumes when bound to prothrombinase. The results reported here shed new light into some of these unresolved issues and set the stage for future analysis.

Proteolysis by chymotrypsin at W468 in the flexible autolysis loop is a relevant reporter of the conformation of prothrombin and its derivatives meizothrombin, prethrombin-2 and thrombin (Fig. 1). Specifically, the open form is cleaved at W468 but the closed form is not, which suggests the following scenario for the transition of prothrombin to thrombin. We propose that under conditions where activation proceeds along the meizothrombin pathway (cleavage at R320 first, followed by cleavage at R271), prothrombin exists predominantly in the closed conformation that is not susceptible to chymotrypsin proteolysis in the autolysis loop, promotes cleavage at R320 and generates meizothrombin that switches to the open form and exposes R271. Meizothrombin bound to prothrombinase remains in the open form and promotes the second cleavage at R271. This generates thrombin with a conformation of the autolysis loop that is essentially identical to that of meizothrombin and susceptible to chymotrypsin proteolysis. We also propose that under conditions where activation proceeds along the prethrombin-2 pathway (cleavage at R271 first, followed by cleavage at R320), prothrombin binds to prothrombinase in the open form, exposes R271 to cleavage and generates prethrombin-2. Removal of the Gla domain and kringles simplifies the conformational flexibility of prethrombin-2 that exposes R320 for a second cleavage and generates thrombin. The meizothrombin pathway of activation involves a conformational of switch in the multi-domain architecture of the prothrombinase substrates, which most likely requires participation of prothrombinase itself through a conformational transition specifically tailored to the open and closed forms. The possibility of prothrombinase existing in alternative conformations has been proposed by Nesheim^7,8^. Likewise, alternative conformations of prothrombin have been postulated by Krishnaswamy^10,14^ long before structural identification of the open and closed forms by our group^16–18^. A scenario that merges these previous proposals is more realistic and is definitely supported by the result presented in this study. Further support comes from the extended surface of recognition between prothrombin and prothrombinase suggested by molecular models^29,30^ and mutagenesis. Kringle-1 and kringle-2 interact with cofactor Va^24,25^ and so do residues within the protease domain scattered between the autolysis loop^31^ and exosite I^32^. Factor Xa interacts with kringle-2^26^ and residues near exosite II of the protease domain^33^, as well as with the Gla domain^34^. Extensive involvement of structural determinants makes both prothrombin and prothrombinase major drivers of the interaction.

The intriguing properties of the R296A mutant deserve attention in this context. The mutant exists in an open-like form susceptible to chymotrypsin cleavage and is activated along the prethrombin-2 pathway. A single amino acid substitution alters the open-closed equilibrium of prothrombin and presents the sites of cleavage to prothrombinase in a different order. Indeed, R296 is positioned right in the middle of R271 and R320 and can provide a key structure-function link that dictates the order in which the sites are presented to prothrombinase and their connection with the allosteric open-closed equilibrium. The results are of mechanistic relevance and also explain the pathological consequences^27^ of disrupting the important role of R296 in prothrombin allostery.

## Methods

### Reagents

Prothrombin wild-type, mutants R296A, Y93A (open), S101C/A470C (closed), truncated forms (i.e., ΔGla, ΔK1, ΔK2, ΔLnk1, ΔLnk2, ΔLnk3), meizothrombin, prethrombin-2 and factor Xa were cloned in a pNUT vector, expressed in BHK cells and purified by affinity chromatography, ion exchange chromatography, and size exclusion chromatography as previously described^16–19^. Homogeneity and chemical identity of final preparations were verified by SDS-PAGE and N-terminal sequencing. Small unilamellar vesicles composed of phosphatidylcholine and phosphatidylserine in a 3:1 molar ratio (PC:PS 75:25) were prepared by extrusion using 100 nm polycarbonate membranes (Avanti Polar Lipids, AL) and their size was confirmed by Dynamic Light Scattering. The vesicles were kept a 4 °C and used within 2 weeks. Human factor Xa, human cofactor Va and dansylarginine-N-(3-ethyl-1,5-pentanediyl) amine (DAPA) were purchased from Hematological Technologies (VT). The chromogenic substrate H-D-Phe-Pro-Arg-p-nitroanilide (FPR-pNA) was from Bachem. All other chemicals were purchased from Sigma-Aldrich (MO).

### Limited proteolysis

Proteins (0.1 mg/ml) were reacted with chymotrypsin (Promega, WI) at 1:50 (w/w) ratio in TBS buffer (10 mM Tris, 145 mM NaCl, 5 mM CaCl_2_, pH 7.4) at 37 °C. At different time intervals (0-5-15-30-60-90-120 min), aliquots of proteolysis mixtures were quenched with NuPAGE LDS buffer and analyzed by non-reducing SDS-PAGE (4–12% acrylamide). Chemical identities of proteolytic products were verified by N-terminal sequencing (see Supplementary Table S1). After Coomassie stained, electrophoretic bands were electrotransferred onto Immobilon-P membrane (Millipore, MA) and chemically characterized by Edman degradation on a Procise-491 automated protein sequencer from Applied Biosystems (MA). None of the gels were cropped and originals are available in the Supplementary Information.

### Prothrombin activation

Prothrombin activation was monitored by SDS-PAGE in the presence of 60 μM DAPA in TBS buffer at 37 °C, using prothrombinase composed of factor Xa (0.5 nM), PC:PS vesicles (50 μM) and cofactor Va (30 nM)^18^. At different time intervals (0-2-5-10-30-120-180 min), aliquots of reactions mixture were quenched with NuPAGE LDS buffer with β-mercaptoethanol as reducing agent and analyzed by SDS-PAGE (4–12% acrylamide). Activation of prothrombin and its truncated forms by prothrombinase (50 pM factor Xa, 50 μM PC:PS vesicles, 30 nM cofactor Va) were determined in a discontinuous assay using a chromogenic assay as detailed elsewhere^18^. Different concentrations of prothrombin (0–1 μM, serial dilution 1:2) were activated by factor Xa in the presence of PC:PS vesicles and cofactor FVa. After 0.5, 1, 2, 3 and 5 min, aliquots were quenched with 20 mM EDTA and active site generation was quantified in terms of the hydrolysis of FPR-pNA (80 μM), specific for thrombin and meizothrombin. Initial velocities were converted to concentrations using a standard reference curve measured at the time of the experiment. Data were collected on a SpectraMax microplate reader (Molecular Devices, CA) and analyzed with Origin 2015 (OriginLab Corp., MA).

## Supplementary information

Supplementary Material

## Data Availability

Reagents and data presented in this study are available from the corresponding author upon reasonable request.
