# Supplementary material for: Probing prothrombin structure by limited proteolysis
Source: Sci Rep. 2019 Apr 16;9:6125. doi: 10.1038/s41598-019-42524-z (PMC6467981; doi:10.1038/s41598-019-42524-z)
Supplement: Supplementary file 1 — Supplementary Material [file 41598_2019_42524_MOESM1_ESM.pdf]

SUPPLEMENTARY MATERIAL

Probing prothrombin structure by limited proteolysis

Laura Acquasaliente, Leslie A. Pelc and Enrico Di Cera

Edward A. Doisy Department of Biochemistry and Molecular Biology, Saint Louis University School of Medicine, St. Louis, MO 63104 USA

Raw gels in Figure 2, 3, 4 and 5. Supplemetary Table listing all N-terminal sequencing results.

Figure S2

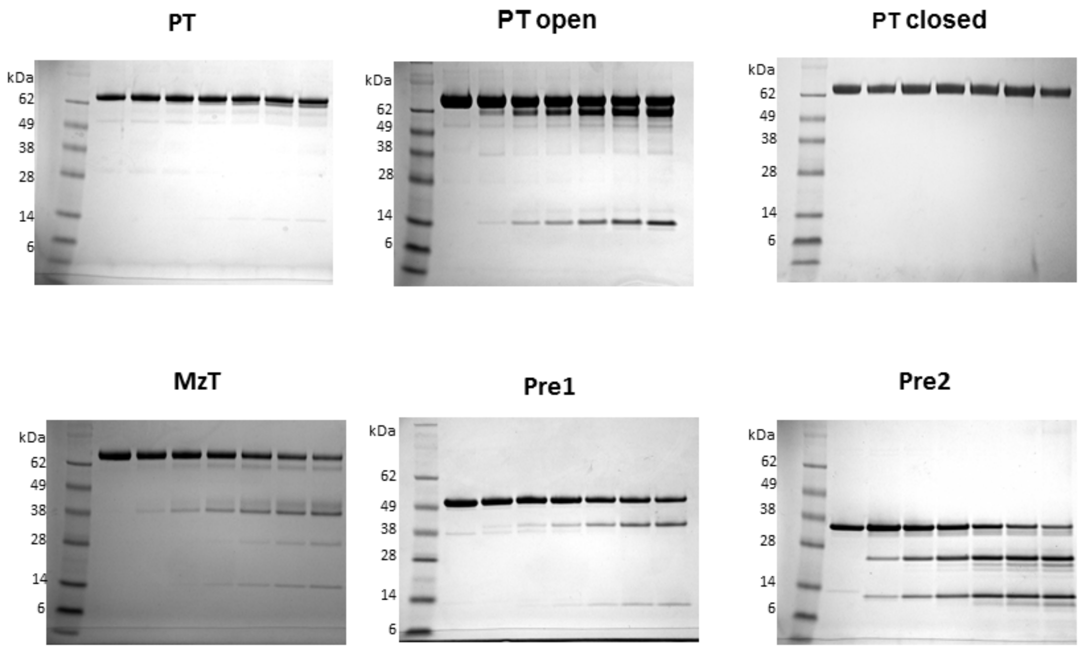

Figure S3

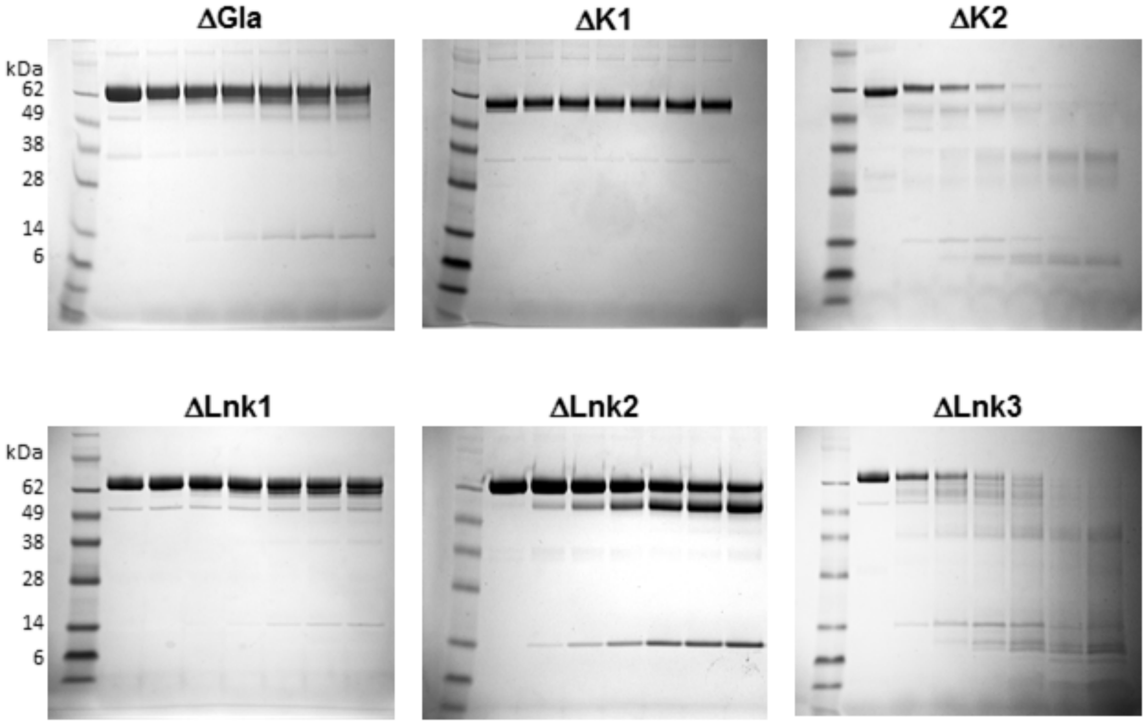
ΔGla
ΔK1
ΔK2
kDa
62
49
38
28
14
6
ΔLnk1
ΔLnk2
ΔLnk3
kDa
62
49
38
28
14
6

Figure S4

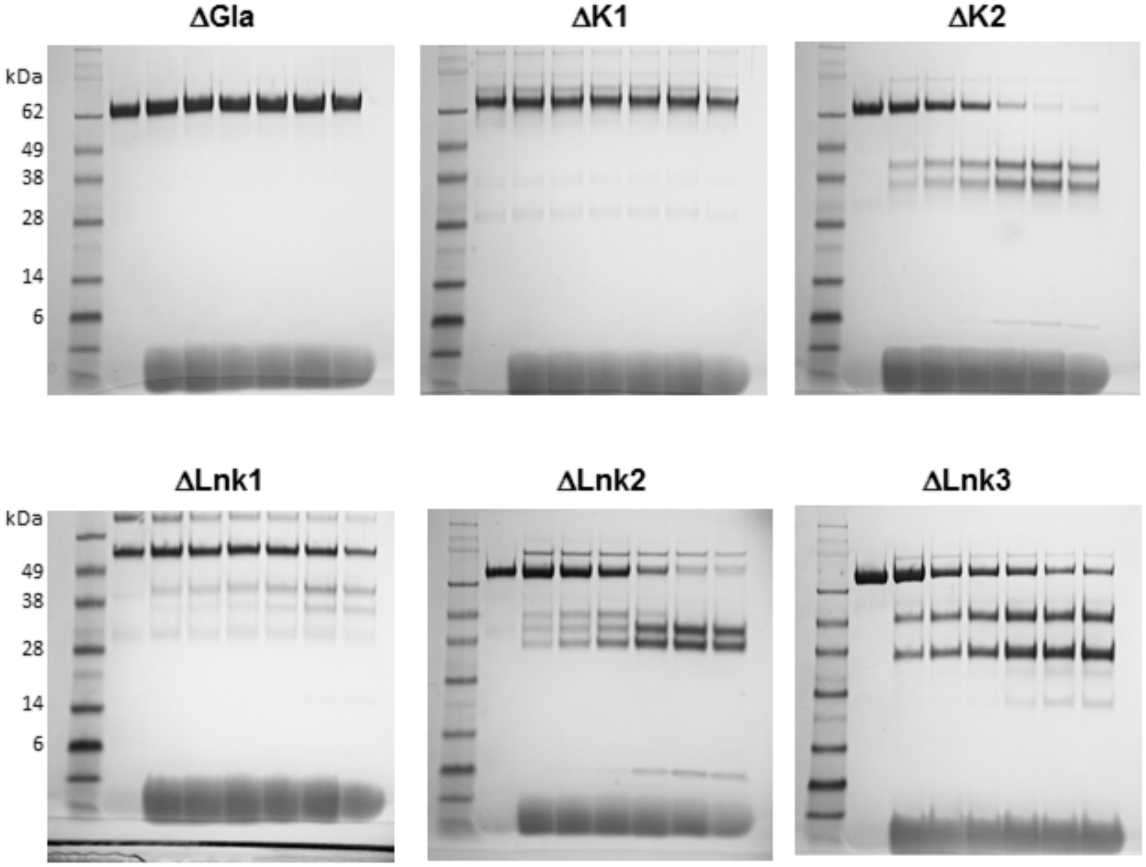
ΔGla
ΔK1
ΔK2
kDa
62
49
38
28
14
6
ΔLnk1
ΔLnk2
ΔLnk3
kDa
49
38
28
14
6

Figure S5

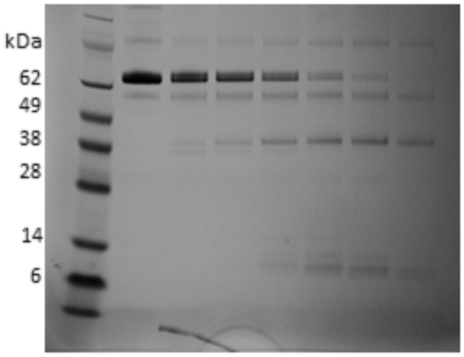
kDa
62
49
38
28
14
6

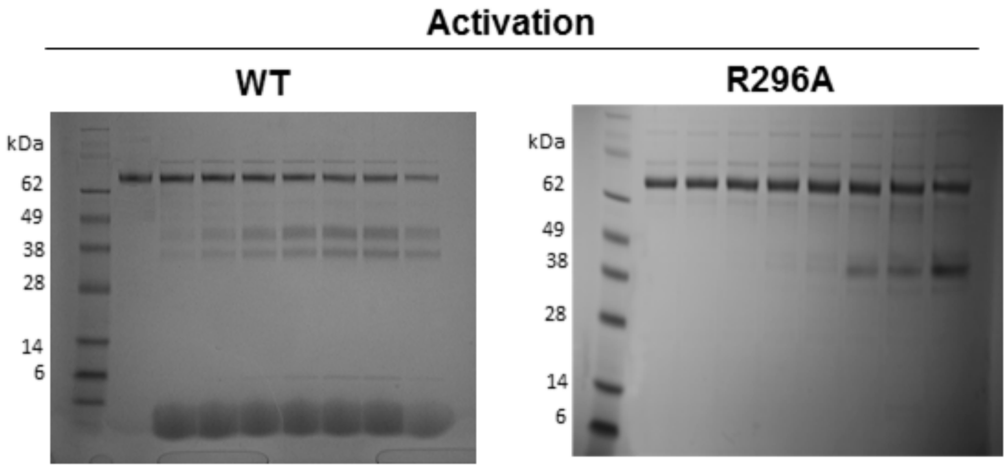
Activation
WT
R296A
kDa
62
49
38
28
14
6
kDa
62
49
38
28
14
6

Table S1. Edman degradation of N-terminal sequences of proteolytic peptides

Protein	Band no.	Size (kDa)	N-terminal sequence	Peptide (cleavage site)	Theoretical mass value (kDa)*

Chymotrypsin digestion

Protein	Band no.	Size (kDa)	N-terminal sequence	Peptide (cleavage site)	Theoretical mass value (kDa)*
PT	1	62	ANTFL	-	65.3
MzT	1	62	ANTFL	-	65.3
	2	55	ANTFL	1-468 (↓W468)	52.8
	3	38	QTF_N	278-579 (↓Y277)	34.7
	4	36	NPR__	282-579 (↓F281)	34.1
	5	26	ANTFL<br>QTF *(minor specie)*	F1<br>278-468	17.3<br>22.2
	6	10	TANVG	469-579 (↓W468)	12.5
Pre2	1	36	TFGS	-	33.8
	2	17	GEAD	289-468 (↓W468)	21.2
	3	12	TANVG	469-579 (↓W468)	12.5
Pre1	1	51	SEGSS	-	48.0
	2	38	SEGS_	156-468 (↓W468)	35.5
	3	10	TANV	469-579 (↓W468)	12.5
PT open	1	62	ANTFL	-	65.3
	2	55	ANTFL	1-468 (↓W468)	52.8
	3	10	TANV_	469-579 (↓W468)	12.5
ΔGla	1	60	CAEG	-	58.2
ΔK1	1	55	ANTFL	-	55.7
ΔK2	1	60	ANTFL	-	55.6
	2	50	ANTFL	1-468 (↓W468)	52.8
	3	28	SPP__	164-468 Δ168-249 (↓L163)	26.2
	4	5	IH_RY	406-468 (↓Y405)	7.5
ΔLnk1	1	62	ANTFL	-	62.8
ΔLnk2	1	62	ANTFL	-	62.5
	2	12	TANVG	469-579 (↓W468)	12.5
ΔLnk3	1	62	ANTFL	-	61.2
	2	50	ANTF_	1-468 (↓W468)	52.8
	3	36	SP_LE	164-468 Δ250-282 (↓L163)	31.0
	4	5	IHP__	406-468 (↓Y405)	7.5
PT R296A	1	62	ANTFL	-	65.3
	2	40	SKQ__<br>ANTFL	203-479 (↓L202)<br>1-357 (↓W357)	43.0<br>39.7
	3	30	VLTA<br>QTF *(minor specie)*<br>NPR_F *(minor specie)*	358-579 (↓W357)<br>278-579 (↓Y277)<br>282-579 (↓F281)	26.5<br>34.7<br>34.1

PTase Activation

Protein	Band no.	Size (kDa)	N-terminal sequence	Peptide (cleavage site)	Theoretical mass value (kDa)*
PT	1	62	ANTFL	-	65.3
	2	40	-	F1.2 A-chain	35.6

	3	30	-	B-chain	29.7
PT R296A	1	62	ANTFL	-	65.2
	2	38	TATS	Pre2	35.2
ΔGla	1	60	CAEG	-	58.2
ΔK1	1	55	ANTFL	-	55.7
ΔK2	1	60	ANTFL	-	55.6
	2	38	TAT_E	Pre2	35.2
	3	30	IVEG	B-chain	29.7
ΔLnk1	1	62	ANTFL	-	62.8
	2	36	ANTF	F1.2	30.8
	3	30	-	B-chain	29.7
ΔLnk2	1	62	ANTFL	-	62.5
	2	40	-	F1.2/Pre2	30.8/35.2
	3	30	IVE	B-chain	29.7
ΔLnk3	1	62	ANTFL	-	61.2
	2	50	SEGS	Pre1	48.0
	3	30	-	B-chain	29.7

*The theoretical mass values were calculated based on the amino acid composition.
